# Predictors of Support for Euthanasia and Physician-Assisted Suicide (EPAS) Among Older Adults in Israel

**DOI:** 10.3390/ejihpe15100207

**Published:** 2025-10-11

**Authors:** Amit Dolev Nissani, Norm O’Rourke, Sara Carmel, Yaacov G. Bachner

**Affiliations:** 1 Goldman School of Medicine, Faculty of Health Sciences, Ben-Gurion University of the Negev, P.O. Box 653, Be’er Sheva 8410501, Israel; amitdol@post.bgu.ac.il; 2 Department of Epidemiology, Biostatistics, and Community Health Sciences, Faculty of Health Sciences, Ben-Gurion University of the Negev, P.O. Box 653, Be’er Sheva 8410501, Israel; orourke@bgu.ac.il; 3 The Center for Multidisciplinary Research in Aging, Faculty of Health Sciences, Ben-Gurion University of the Negev, P.O. Box 653, Be’er Sheva 8410501, Israel; sara@bgu.ac.il; 4 Department of Psychology, Faculty of Humanities and Social Sciences, Ben-Gurion University of the Negev, P.O. Box 653, Be’er Sheva 8410501, Israel

**Keywords:** euthanasia, Israel, older adults, perceived control, physician-assisted suicide, religiosity, self-efficacy, social support

## Abstract

Background: Euthanasia and physician-assisted suicide (EPAS) are highly contentious topics with significant medical, legal, and cultural implications. Previous research suggests that various sociodemographic, health, and psychosocial factors determine attitudes toward EPAS. This study set out to identify psychosocial predictors of support for EPAS. We hypothesized that perceived control, self-efficacy, and social support would each predict support for EPAS after controlling for sociodemographic and health-related variables. Methods: For this study, we recruited 446 Jewish Israeli adults who were 82.32 years of age on average (SD = 5.99; range 65–101 years). Participants completed a battery of questionnaires including a series of vignettes featuring hypothetical family members with a terminal illness (i.e., cancer, dementia, Parkinson’s disease). We performed a three-step hierarchical regression equation, controlling for demographic factors (age, gender, education, relationship status, economic status, and religiosity) as well as perceived and relative physical health. Results: As hypothesized, both self-efficacy and (the absence of) social support predicted support for EPAS; perceived control did not. Religiosity was the strongest predictor of opposition to EPAS. Fully 31% of variance in support for EPAS was predicted by this regression model. Conclusion: Support for EPAS does not appear to reflect a pervasive need for control over all aspects of life (i.e., perceived control) but a more specific need for personal autonomy (i.e., self-efficacy). Longitudinal research is required over multiple points of data collection to ascertain how change in social support affects support for EPAS in late life. Policy makers should embrace these findings when formulating end-of-life care policies, ensuring that both social support and personal autonomy are prioritized in the care of older adults.

## 1. Introduction

Euthanasia, or voluntary active euthanasia, is defined as a deliberate act of terminating a person’s life at their explicit request in order to prevent further suffering (Marina et al., 2022; Mimarakis et al., 2025). By contrast, physician-assisted suicide is where a medical doctor provides medication or a prescription to the patients at their explicit request, with the understanding that patients intend to use the medication to end their life (Emanuel et al., 2016; Kono et al., 2023; Marina et al., 2022; Picón-Jaimes et al., 2022). From a clinical perspective, the difference is that in euthanasia the physician administers the intervention, whereas in physician-assisted suicide the patient does so. Ethically, this distinction emphasizes patient autonomy versus physician responsibility in end-of-life care.

Existing research indicates that various sociodemographic variables are associated with attitudes toward euthanasia and physician-assisted suicide (EPAS) (Inglehart et al., 2021). Education and religiosity have repeatedly emerged as predictors of support for EPAS (Karumathil & Tripathi, 2022; Castelli Dransart et al., 2021; Mimarakis et al., 2025). That is, more highly educated people are inclined to favor physician assisted suicide compared to those with less education (Bulmer et al., 2017; Castelli Dransart et al., 2021). Religiosity is widely recognized as the strongest predictor of opposition to euthanasia; secular people are more likely to favor actively ending one’s life compared to religious people (Castelli Dransart et al., 2021; Guzowski et al., 2024; Mimarakis et al., 2025). This has been found with adherents of Abrahamic religions (i.e., Christianity, Judaism and Islam; Aghababaei et al., 2014) and Hinduism (Grove et al., 2022).

Other sociodemographic factors also predict EPAS, though less consistently. Age appears to be inversely associated with support for EPAS, as younger people generally hold more liberal attitudes than older adults (Karumathil & Tripathi, 2022; Castelli Dransart et al., 2021; Mimarakis et al., 2025). Gender is also associated with support for EPAS as men are more likely to support EPAS than women (Aghababaei & Wasserman, 2013; Castelli Dransart et al., 2021). Associations between support for EPAS, marital status and socioeconomic status are inconsistent (Castelli Dransart et al., 2021; Karumathil & Tripathi, 2022). Moreover, the association between physical health and support for EPAS is unclear. While some studies report no relationship between physical health and support for EPAS (Scheeres-Feitsma et al., 2023; Stolz et al., 2017b), others suggest that poorer physical health is linked with greater support for EPAS (Castelli Dransart et al., 2021; Kono et al., 2023).

Attitudes towards EPAS are also shaped by factors beyond physical health (Luna-Meza et al., 2021). These can include psychological distress, social isolation, fear of dying, and personal experiences with suffering and loss (Castelli Dransart et al., 2021; Kono et al., 2023; Mimarakis et al., 2025). This implies that individual differences shape attitudes toward end-of-life decisions.

Research asserting a negative association between physical health and support for EPAS contends that those with severe physical illnesses, chronic pain, or substantial declines in quality of life are more likely to support EPAS (Liu et al., 2021; Rahimian et al., 2024). These studies suggest that loss of bodily autonomy, reliance on others for basic self-care, and persistent and uncontrollable chronic pain motivates individuals to make proactive, end-of-life decisions. For these individuals, EPAS is seen as a means to regain and retain dignity and autonomy in the face of debilitating physical conditions (Stolz et al., 2017b; Castelli Dransart et al., 2021). EPAS is a means to reclaim a sense of control by allowing individuals to determine the timing and manner of their own death (Rietjens et al., 2005; Stolz et al., 2017a; Castelli Dransart et al., 2021).

Perceived control over life and health is assumed to be pivotal in shaping attitudes toward EPAS. Perceived control not only influences patients’ desire to hasten death but also their support for legislative measures and ethical frameworks surrounding end-of-life decision making. Research indicates that clinician attitudes toward EPAS are likewise influenced by personal beliefs regarding patient autonomy and control over treatment decisions (Castelli Dransart et al., 2021; Velasco Sanz et al., 2022).

Similarly, self-efficacy is the belief that one is capable of acting in ways necessary to attain specific goals. This construct has been widely studied in relation to health-related behaviors and decision making processes; yet associations with EPAS have received little research focus to date. However, individuals with higher self-efficacy likely exhibit stronger desire to manage their health outcomes and end-of-life decision making. This may well extend to attitudes toward euthanasia, particularly in terms of autonomy and control over medical choices (Jo & Lee, 2008; Lee et al., 2020).

Research examining the role of social support in relation to EPAS is inconsistent (Corcoran et al., 2024). However, supportive relationships provide emotional, practical, and ethical guidance, affecting will to live (Carmel et al., 2023). Moreover, cultural norms and societal values embedded within these networks shape whether individuals perceive these end-of-life options as contentious, morally problematic or a legitimate individual choice (Bakhsh & Yaseen, 2024; Castelli Dransart et al., 2021; Guzowski et al., 2024).

Given the rise in life expectancy and the increasing availability of advanced medical interventions at the end of life, decisions regarding euthanasia and physician-assisted suicide (EPAS) are highly relevant for older adults. However, research examining the psychosocial determinants of EPAS among this population remains limited. Moreover, few studies have examined the Israeli context, where factors such as religiosity, perceived control, and social support may play a particularly important role. In this study, we aimed to identify predictors of support for EPAS among older adults in Israel. We hypothesized that perceived control, self-efficacy, and social support would each predict support for EPAS after controlling for sociodemographic and health-related variables. A clearer understanding of the specific factors associated with EPAS in this population may inform evidence-based policy and guide clinical and psychological practice in end-of-life-care.

## 2. Materials and Methods

### 2.1. Sample and Population

For this cross-sectional study, we recruited a convenience sample of 466 older Jewish adults living independently in all regions of Israel (i.e., north, south and center). We selected those independent in their ability to perform activities of daily living and 65+ years of age. Participants were recruited from day centers, community organizations and word-of-mouth (i.e., snowball strategy). We excluded those under 65, living in institutions, and diagnosed with neurological conditions (e.g., dementia).

Almost half of all participants were born in Israel (216 of 466, or 46.4%). The next largest group were Mizrahi Jews (82 of 466, or 17.6%) who fled or were expelled from Arab countries after 1948 (i.e., Egypt, Syria, Iraq), followed by emigrants from the former USSR who arrived after 1989. The smallest group were Holocaust survivors (42 or 466, or 9%). Remaining participants came from India, Ethiopia, South Africa, and South America (e.g., Argentina). Most participants were recruited before the COVID-19 pandemic.

We compared participants recruited after March 2020 vis-à-vis those recruited before the pandemic. The former (M = 84.14, SD = 6.60) were somewhat older than participants recruited before the pandemic (M = 82.14, SD = 5.97; t[464] = 2.20, *p* = 0.03). Yet both groups reported similar levels of perceived health (t[464] = 1.27, *p* = 0.21) and relative health, t(464) = 1.08, *p* = 0.28. Moreover, responses to study variables did not differ, including attitudes toward EPAS, t(464) = 0.02, *p* = 0.98. It would not appear that the pandemic significantly effected who was recruited or their responses to study variables. See Table 1.

Participants were 82.35 years of age on average (SD = 6.06), ranging from 65 to 101 years. EPAS is not an esoteric, philosophical issue for older adults, but germane to their lives today.

For this study, we developed a series of vignettes to measure support for EPAS. This methodology is not unique as several such studies have previously been conducted (e.g., Hofmann & Wagner, 2025; Rietjens et al., 2005). Most research however has been conducted in northern Europe where religiosity is low, aside from immigrant communities (Burkimsher, 2014). This introduces the risk of floor effects when studying attitudes toward topics such as EPAS. Israel, in contrast, is considerably more religious than northern Europe. Such research conducted in Israel to date has largely focused on clinicians, not older adults (e.g., Ganz & Musgrave, 2006; Levy et al., 2013).

Religiosity is only one of many ways in which Israel differs from other developed countries. Not only is Israel the only Jewish-majority nation but Israel has the highest rate of fertility of any OECD country; this includes both secular and religious Israelis. As a result the average age of 30.5 years in Israel is considerably lower than most European countries (cf. 40–43 years). Rates of education, urbanization and life expectancy are also high. And even during wartime, Israelis consistently rank among the world’s most happy, similar to northern Europe, but higher than all other Middle Eastern nations (#8 for 2024, Helliwell et al., 2025).

### 2.2. Instruments

**Support for EPAS** was measured by responses to four vignettes involving hypothetical family members. The scenarios pertained to relatives with metastatic cancer (40 and 80 years old), Alzheimer’s and Parkinson’s disease (Lifshitz et al., 2024). For example:

“‘A’ is an immediate family member with metastatic cancer and no chance of recovery. He is 40-years old and has about six months to live. ‘A’ is experiencing severe physical pain and requires assistance to carry out basic tasks. His physician has explained the available options for pain management and the possibility of receiving supportive care to relieve both physical and emotional symptoms (palliative care), either at home or as a hospice inpatient. Instead, ‘A’ has repeatedly asked his doctor to help him end his life due to the suffering caused by cancer”.

For each of four vignettes, participants were asked to indicate their support for both active euthanasia (i.e., “Should the physician administer a lethal dose of medication in order to end your relative’s life?”) and physician-assisted suicide (i.e., “Should the physician provide a lethal dose of medication to your relative so that he can end his/her own life?”). Responses were provided along a Likert scale ranging from *strongly disagree* (1) *to strongly agree* (6). As this vignette methodology has been used repeatedly in EPAS research (e.g., Hofmann & Wagner, 2025; Jingyuan et al., 2025; Takimoto & Nabeshima, 2025), we did not pilot test vignettes.

A small percentage of participants supported euthanasia but not physician-assisted suicide (45 of 466, or 9.7%), and fewer still supported physician-assisted suicide but not euthanasia (11 of 466, or 2.4%). Setting these participants (temporarily) aside, responses to questions regarding active euthanasia (four items) and physician-assisted suicide (four items) were strongly correlated across vignettes (r = 0.87, *p* < 0.01); this suggests measurement of a single construct and supports our decision to combine responses for all vignettes. Internal consistency of responses was high (EPAS–Family, α = 0.92).

**Social support** was measured using the 12-item Multidimensional Scale of Perceived Social Support (MSPSS; Zimet et al., 1988), which examines support from family, friends, and others (e.g., “My family really tries to help me”). Respondents are provided along a Likert type scale ranging from *strongly disagree* (1) to *strongly agree* (7).

**Self-efficacy** was measured using the General Self-Efficacy Scale (GSES) translated to Hebrew by Schwarzer and Jerusalem (1995) for use in Israel. The scale includes 10 items measuring perceived abilities under various situations (e.g., “I am confident that I can handle unexpected events”). Respondents are provided along a Likert type scale ranging from *not at all true* (1) to *exactly true* (4).

**Perceived control** was measured using the scale developed for use with older adults by Pearlin and Schooler (1978). The scale includes 7 items assessing individual’s belief in their ability to influence life events and outcomes (e.g., “What happens to me in the future mostly depends on me”). Responses are provided along a Likert type scale ranging from *strongly disagree* (1) to *strongly agree* (5).

**Perceived health** status was measured by the item “overall, how is your health; and **relative health** by the item “how is your health compared to others your age?”. Responses to both items were provided on a Likert scale ranging from *very poor* (1) to *excellent* (6).

**Sociodemographic characteristics** included age (in years), gender (male/female), marital status (married/divorced/single/widowed), number of children, country of birth (Israel/other), level of education (primary/partial secondary/secondary/post-secondary/partial academic/academic), and economic status (above or below national average). Level of Jewish religiosity was measured as: secular, traditional (observes some commandments), religious (observes most commandments), very religious (observes all commandments), and ultraorthodox (i.e., Haredi). This continuum applies to both Ashkenazi and Mizrahi Jews in Israel.

### 2.3. Data Analysis

Descriptive statistics are reported for continuous study variables (means, standard deviations, ranges). Associations between variables were analyzed using Pearson, Spearman, or chi-square tests according to scale structures. The unique contribution of psychosocial variables to the explanation of the EPAS variance was assessed using hierarchical linear regression. With 466 participants and 11 independent variables, this regression equation had sufficient statistical power to identify medium to small effect sizes (α < 0.05; Cohen, 2013). Data analyses were performed using SPSS, version 29.

## 3. Results

Table 2 presents descriptive statistics for the continuous study variables. The mean level of approval for EPAC-Family was moderate (*M* = 22.17, *SD* = 12.15), considering the possible scale range (8–48). In contrast, participants reported relatively high levels of perceived control, self-efficacy, and social support. Internal consistency of responses to all scales was in ideal parameters (i.e., 0.80 ≤ α ≤ 0.93).

Table 3 reports correlation coefficients between continuous study variables. Age is negatively correlated with EPAS-Family (r = −0.13, *p* = 0.01), perceived control (r = −0.14, *p* = 0.01) and self-efficacy (r = −0.16, *p* = 0.01), but not social support (r = −0.01, *p* = 0.87). Perceived control is strongly correlated with self-efficacy (r = 0.54, *p* = 0.01) and moderately correlated with social support (r = 0.27, *p* = 0.01). Similarly, self-efficacy is moderately correlated with social support (r = 0.34, *p* = 0.01). Of note, EPAS-Family is significantly correlated only with self-efficacy (r = 0.12, *p* = 0.01).

To determine the unique contribution of the variables predicting support for EPAS, a 3-step hierarchical regression analysis was conducted. This allowed us to determine the unique variance explained by first health variables (perceived and relative health) then, individual differences variables (self-efficacy, perceived control, social support) after covarying for sociodemographic variables (i.e., age, gender, education, religiosity, relationship and socioeconomic status). See Table 4.

In the second step, health variables were added to the model to examine their unique contribution to prediction beyond demographic factors. Neither perceived nor relative health were found to be significant. Health variables explained only 1% of unique variance in support for EPAS, Δ*R*^2^ = 0.01, *p* < 0.01.

In the third and final step, self-efficacy (β = 0.11, *p* = 0.03) and lower social support (β = −0.10, *p* = 0.02) both emerged as significant predictors of support for EPAS; in contrast, perceived control did not (β = −0.01, *p* = 0.82). This step accounted for just a further 1% of unique variance; as hypothesized, psychosocial variables emerged as significant predictors of support for EPAS over-and-above both health and sociodemographic variables, including religiosity.

## 4. Discussion

For this study, we set out to identify psychosocial predictors of support for EPAS among older adults, over-and-above sociodemographic and health variables. Average support for EPAS was found to be moderate in this sample (M = 22.17, range 8–48). This reflects the national sentiment in Israel where there is no groundswell to legalize EPAS. Under the Dying Patient Law (Israel, 2005), patients can legally refuse or discontinue certain medical interventions and provide advanced directives (e.g., do not resuscitate). However, active euthanasia and physician-assisted suicide remain prohibited by law.

In contrast, most research conducted in other developed countries tends to report higher levels of support for EPAS among older adults. For instance, a cross-national study of 62 countries found that those who support for euthanasia, particularly in high-income countries, tend to be less religious. Highest support for euthanasia was reported in the Netherlands (Inglehart et al., 2021) where 47% of respondents expressed support for euthanasia or physician-assisted suicide for a family member with dementia (Scheeres-Feitsma et al., 2023).

The findings of the regression analysis shed light on the complex network of sociodemographic, health-related, and psychosocial factors shaping attitudes toward euthanasia and physician-assisted suicide (EPAS) among older Jewish adults in Israel. Consistent with previous literature, religiosity emerged as the strongest and most consistent predictor of opposition to EPAS. Participants who identified as more religious were significantly less likely to support EPAS, reinforcing prior findings that religious frameworks—especially within traditional Jewish contexts (i.e., ultraorthodox)—tend to emphasize the sanctity of life over individual autonomy (Castelli Dransart et al., 2021; Guzowski et al., 2024; Mimarakis et al., 2025).

Education did not emerge as a significant predictor of EPAS in contrast to previously published research. Generally, higher education is associated with more liberal views on ethical and medical autonomy (Bulmer et al., 2017; Castelli Dransart et al., 2021). Female participants were found to be more supportive of EPAS than males, a finding that contrasts with some earlier studies suggesting greater male endorsement of assisted dying (Aghababaei & Wasserman, 2013; Castelli Dransart et al., 2021). This discrepancy may reflect changing societal roles or gendered experiences of caregiving and suffering in late life, warranting further exploration.

Surprisingly, age did not significantly predict support for EPAS. This contradicts prior studies that identified younger age as a correlate of increased support for assisted dying (Castelli Dransart et al., 2021; Karumathil & Tripathi, 2022; Mimarakis et al., 2025). One possible explanation lies in the homogeneity of the current sample, which comprised relatively old participants (M = 82.35), all living independently. Within this age band, variation in attitudes may be more strongly shaped by internal resources (e.g., coping style, social support) than by age per se. Furthermore, the absence of younger adults in the sample limits direct comparison across cohorts.

Health status, both perceived and relative health, was not found to be a significant predictor of support for EPAS—contributing just 1% of unique variance. This finding diverges from studies linking poorer physical health to greater support for euthanasia (Castelli Dransart et al., 2021; Stolz et al., 2017a). It may be that subjective perceptions of suffering, autonomy, or existential distress—rather than objective health status—play a more central role in shaping end-of-life attitudes among older adults. Moreover, the selection criteria of this study, which required participants to be functionally independent, may have reduced variance in physical health and, by extension, its predictive utility (Jo & Lee, 2008; Lee et al., 2020).

Conversely, lower levels of social support predicted increased support for EPAS. This echoes previous work suggesting that social isolation and lack of emotional connection may fuel the desire to maintain control over one’s dying process (Castelli Dransart et al., 2021; Karumathil & Tripathi, 2022; Guzowski et al., 2024). Notably, perceived control—though conceptually related to autonomy—was not a significant predictor. This finding underscores the nature of support for EPAS by older adults. It is not a general need to control over one’s life but a need for personal autonomy that appears to determine support for EPAS in late life.

Literature suggests that, particularly among older adults, social support is often rooted in family relationships. Our results indicate that those perceiving lower social support (including from family) were more likely to endorse EPAS for an ill family member.

This stands in contrast to a recent meta-analysis by Corcoran et al. (2024), which found no association between social connectedness and EPAS. Future studies should explore whether family-focused vignettes, such as those used in our study, elicit different responses compared to generic patient scenarios. longitudinal research is needed to clarify these patterns.

The above findings reinforce the understanding that support for euthanasia and physician-assisted suicide (EPAS) is not solely determined by sociodemographic variables such as religiosity, but rather emerges from a complex interplay of psychological, social, and cultural factors. While sociodemographic variables accounted for a substantial portion of the variance, it is equally important to explore individual-level predictors that shape end-of-life attitudes. In the present study, higher levels of self-efficacy were associated with greater support for EPAS, whereas lower levels of social support predicted increased endorsement. Importantly, by situating this research in the Israeli context—where EPAS remains prohibited and religious traditions strongly emphasize the sanctity of life—we were able to demonstrate how cultural frameworks intersect with psychosocial factors in shaping attitudes. Moreover, our use of family-focused vignettes shed light on the central role of familial relationships in older adults’ ethical decision making, a dimension that may be overlooked in studies relying on more generic patient scenarios. Together, these contributions highlight the influence of personal agency, social isolation, and cultural context on support for EPAS, underscoring the need for future research to examine additional psychosocial variables that could deepen our understanding of the psychological foundations underlying attitudes toward end-of-life care.

### 4.1. Limitations

Several limitations of this study should be acknowledged. First, the sample consisted exclusively of older, functionally independent Jewish Israeli adults, limiting the generalizability of the findings to other populations, including non-Jewish groups or individuals residing in institutional care. Nor did we screen for cognitive loss.

However, recruitment of just Jewish Israelis is both a study strength and limitation. From a psychometric standpoint, measurement of religiosity within a single, heterogeneous religious group is more reliable and valid than across multiple, disparate religions. Had we including Arab Israelis in our sample (i.e., Muslim, Christian, Druse), this would have increased generalizability of findings but complicated measurement of religiosity. For instance, there is no equivalent in Christianity or Islam to ‘traditional Jews’ who are observant but not religious. The continuum of what constitutes religious observance and behavior differs, even across Abrahamic religious (cf. jihad, rosary/stations of the cross). Data collection with an exclusively Jewish sample enabled us to effectively control for the effects of (Jewish) religiosity on support for EPAS within a heterogeneous (Jewish) Israeli sample. We do not contend that our sample is representative. Randomly selected participants should be recruited in order to replicate study findings.

That noted, the results of this study need to be replicated with Muslim and other religious minorities in Israel; the former tend to be even more religious than Israeli Jews (Feldman, 2020). When surveyed, Israeli Muslims (68%), Christians (57%), and Druze (49%) are each more likely than Jews (30%) to say that religion is very important (Pew Research Center, 2016). Comparative research across groups should also be undertaken.

More importantly, this was a cross-sectional study which precludes conclusions about causality or changes in attitudes over time. Finally, data collection was completed during the COVID-19 pandemic—a period characterized by heightened exposure to illness, death, and isolation. Comparative analyses suggest that those recruited before March 2020 were demographically similar to those recruited during the pandemic; nor do responses significantly differ. At least during the first months of the pandemic (i.e., first lockdown), attitudes toward EPAS do not appear to have shifted.

The results of this study suggest that psychosocial variables predict attitudes toward EPAS over-and-above socio-demographic and health variables. Both self-efficacy and (the absence of) social support predict support for EPAS for an ill family member after correcting for gender and religiosity. In contrast, perceived control appears unrelated to support for EPAS.

### 4.2. Directions for Future Research

However, future research is needed in which perceived control is measured with greater focus (e.g., health locus of control). Interaction effects between social support and gender, and self-efficacy and religiosity should be explored. Moreover, we should determine who and why some older adults support euthanasia but not physician-assisted suicide and conversely, and more inexplicably, physician-assisted suicide but not euthanasia.

This research should be conducted over multiple points of measurement to ascertain if fluctuations in social support over time predict change in support for EPAS. This should include more diverse samples (e.g., institutionalized persons) and mixed methods to uncover personal narratives and meaning that underly such complex beliefs and attitudes.

## 5. Conclusions

This study contributes to the growing body of literature examining attitudes toward euthanasia and physician-assisted suicide (EPAS). Our findings underscore the central role of religiosity, yet psychosocial factors also emerged as predictors of support for EPAS after statistical control for sociodemographic and health variables. As public debate around end-of-life options continues, individual differences such as self-efficacy and social support in later life should be taken into account. Comparative, longitudinal research is needed to replicate these findings with other samples and populations (e.g., Jews in the diaspora vs. Israel).

Policy makers should embrace these findings when formulating end-of-life care policies, recognizing the importance of integrating both social support systems and respect for personal autonomy. In practice, this may involve embedding psychosocial assessments into palliative and geriatric care, expanding access to counseling and caregiver support, and training healthcare professionals to engage constructively with diverse cultural and religious perspectives on euthanasia. Such measures can ensure that decisions around end-of-life care are not solely framed by medical or legal considerations but are also responsive to the lived experiences, values, and social support structures of older adults and their families. By embedding these elements into policy and clinical practice, decision makers can create more compassionate, equitable, and ethically robust frameworks that safeguard dignity and well-being in late life while also providing clearer guidance for healthcare professionals navigating these sensitive situations.

## Figures and Tables

**Table 1:** Sociodemographic Features of Study Participants (*N* = 466).

		N	%	Mean	SD
Age	65–101			82.35	6.06
Gender	Male	209	44.80%		
	Female	257	55.20%		
Family Status	Married	250	53.60%		
	Single	5	1.10%		
	Divorced	39	8.40%		
	Widow	166	35.60%		
	Other	6	1.30%		
Num. of children	0–11			3.78	2.14
Country of birth	Israel	211	45.90%		
	Other	249	54.10%		
Education	Primary education	93	21.00%		
	Partial secondary education	43	9.70%		
	Completed secondary education	70	16.00%		
	Post-secondary non-academic education	65	14.70%		
	Partial academic education	30	6.70%		
	Completed academic degree	141	31.90%		
Employment status	Full-time employment	44	9.90%		
	Part time employment	15	3.40%		
	Unemployed	36	8.10%		
	Retired	347	78.00%		
	Other	3	0.60%		
Level of religiosity	Secular	200	43.20%		
	Traditional	91	19.60%		
	Observant	87	18.80%		
	Orthodox	36	7.80%		
	Ultra-orthodox	49	10.60%		
Economic Status	Below average	64	13.70%		
	Average and above	402	86.30%		
Living Arrangement	Living alone	103	23.80%		
	Spouse	253	58.40%		
	Child	46	10.60%		
	Foreign caregiver	24	5.60%		
	Other	7	1.60%		
Perceived Health				4.05	1.06
Relative Health				4.35	1.12

**Table 2:** Descriptive Statistics, continuous variables (*N* = 466).

Variable	Mean (SD)	Range	Skewness	Kurtosis	α
Age	82.35 (6.06)	65–101	0.69	0.52	--
EPAS–Family	22.17 (12.15)	8–48	0.54	−0.55	0.92
Perceived Control	22.92 (5.99)	7–35	−0.04	−0.54	0.80
Self-Efficacy	31.61 (6.48)	10–45	−0.77	0.34	0.93
Social Support	67.52 (14.67)	12–84	−1.19	1.50	0.93

**Table 3:** Correlations among the study measures, continuous variables (*N* = 466).

	EPAS-Family	Perceived Control	Self-Efficacy	Social Support
Age	−0.13 **	−0.14 **	−0.16 **	−0.01
EPAS-Family		0.06	0.12 **	−0.08
Perceived Control			0.54 **	0.27 **
Self-Efficacy				0.34 **

** *p* < 0.01.

**Table 4:** Predictors of Support for EPAS, hypothetical family members (*N* = 466).

Variables	B	SE B	β	*F*
Demographic Features				
Years of age	−0.07	0.09	−0.03	0.60
Gender, 1 = male, 2 = female	2.58	1.05	0.10	5.99 *
Education	0.31	0.21	0.05	1.15
Relationship Status	−0.12	0.38	−0.01	0.09
Economic Status	0.56	0.55	0.05	1.03
Religiosity	−4.37	0.37	−0.51	140.21 **
Physical Health				
Perceived Health	−1.30	0.75	−0.11	2.96
Relative Health	0.26	0.69	0.02	0.14
Individual Differences				
Perceived Control	−0.02	0.10	−0.01	0.05
Self-Efficacy	0.22	0.10	0.11	4.60 *
Social Support	−0.09	0.04	−0.10	5.56 *

* *p* < 0.05, ** *p* < 0.01. Note. *R*^2^ = 0.29 for demographic features, *p* < 0.01; Δ*R*^2^ = 0.01 subsequent to inclusion of health variables, *p* < 0.01; Δ*R*^2^ = 0.01 subsequent to inclusion of individual differences, *p* < 0.01.

## Data Availability

Data supporting reported results can be found by contacting the corresponding author.

## Abbreviation

The following abbreviation is used in this manuscript:

EPAS	Euthanasia and physician-assisted suicide
